# Autoimmune Encephalitis With Multiple Auto-Antibodies With Concomitant Human Herpesvirus-7 and Ovarian Teratoma: A Case Report

**DOI:** 10.3389/fmed.2021.759559

**Published:** 2022-02-14

**Authors:** Jianhua Yang, Pengcheng Wu, Xianghong Liu, Han Xia, Zhaohui Lai

**Affiliations:** ^1^Department of Neurology, The Affiliated Ganzhou Hospital of Nanchang University, Ganzhou, China; ^2^Department of Scientific Affairs, Hugobiotech Co., Ltd., Beijing, China

**Keywords:** human herpes virus 7, anti-N-methyl-D-aspartate receptor (NMDAR) encephalitis, α-amino-3-hydroxy-5-methyl-4-isoxazolepropionic acid receptor (AMPAR) encephalitis, autoimmune encephalitis, post viral encephalitis autoimmune encephalitis, paraneoplastic syndrome

## Abstract

Infectious etiologies and tumors are common triggers of autoimmune encephalitis. We herein reported a rare case of autoimmune encephalitis with multiple autoantibodies in cerebrospinal fluid (CSF) and serum, with concomitant human herpesvirus 7 (HHV-7) infection and ovarian teratoma. A 36-year-old woman presented with mental and behavioral changes and gibberish for 13 days, followed by fever for 1 day. Her brain MRI indicated limbic encephalitis. Metagenomic next-generation sequencing (mNGS) of CSF revealed HHV-7. Antibody testing showed positive anti-N-methyl-D-aspartate receptor (NMDAR) and anti-α-amino-3-hydroxy-5-methyl-4-isoxazolepropionic acid receptor (AMPAR) antibodies in CSF and serum. Ovarian teratoma was considered after pelvic MRI, which was then pathologically confirmed after laparoscopic ovariectomy. Her conditions improved after laparoscopic surgery, intravenous steroids, immunoglobulin, and rituximab therapy. Our findings suggested that the combination of multiple therapies including antiviral, immunotherapy, and resection of tumors were appropriate and improved the prognosis, when HHV-7 infection and ovarian teratoma were concomitant with multiple anti-neuronal antibodies of autoimmune encephalitis.

## Introduction

Human herpesvirus 7 (HHV-7) belongs to Betaherpesvirinae and is ubiquitous, like other human herpesviruses. Primary HHV-7 infection usually occurs during childhood, followed by a lifelong latent state with possible reactivation in case of immunodeficiency (1). The primary infections of HHV-7 can be asymptomatic, or with minor and nonspecific symptoms, including fever, rash, hepatitis, and pneumonitis. The HHV-7 rarely causes encephalitis, especially in immunocompetent patients, although it has a specific tropism for CD4+ lymphocytes and neurons (2). Clinical presentations of HHV-7 infection in the neurological system include a gradual loss of strength and weakness in the limbs, disorientation and confusion, flaccid paraplegia, localized anesthesia and hypoesthesia for pain and light touch, urinary retention, and constipation (3).

Post-viral autoimmune encephalitis has been established as a disease mechanism after herpes simplex virus (HSV) encephalitis. Other viruses including varicella-zoster virus (VZV), Epstein-Barr virus (EBV), human herpesvirus 6 (HHV-6), cytomegalovirus (CMV), adenovirus, enterovirus, HIV, and hepatitis C virus can also cause this disease (4–9). However, there are few reports about this disease that is secondary to HHV-7 (10). The most frequent autoantibody, detected of autoimmune encephalitis is anti-NMDAR antibody, which can cause relapsing symptoms post herpes simplex virus encephalitis (HSE) in up to 28% of HSV encephalitis (11). Other autoantibodies such as anti-gamma-aminobutyric acid receptor, anti-dopamine 2 receptor, and anti-α-amino-3-hydroxy-5-methyl-4-isoxazolepropionic acid receptor (AMPAR) antibodies are also common (8, 12). Coexisting antibodies may produce an overlap of clinical symptoms in patients with autoimmune encephalitis and raise the probability of an underlying malignancy. As is known, autoimmune encephalitis is paraneoplastic, and ovarian teratoma is one of the most common tumors associated with anti-NMDAR encephalitis. It is not common that viral infections and tumor can trigger autoimmune encephalitis simultaneously. We describe here a case of autoimmune encephalitis with anti-NMDAR and anti-AMPAR antibodies in the CSF and serum with concomitant HHV-7 infection and ovarian teratoma.

## Case Presentation

**Figure 1:**
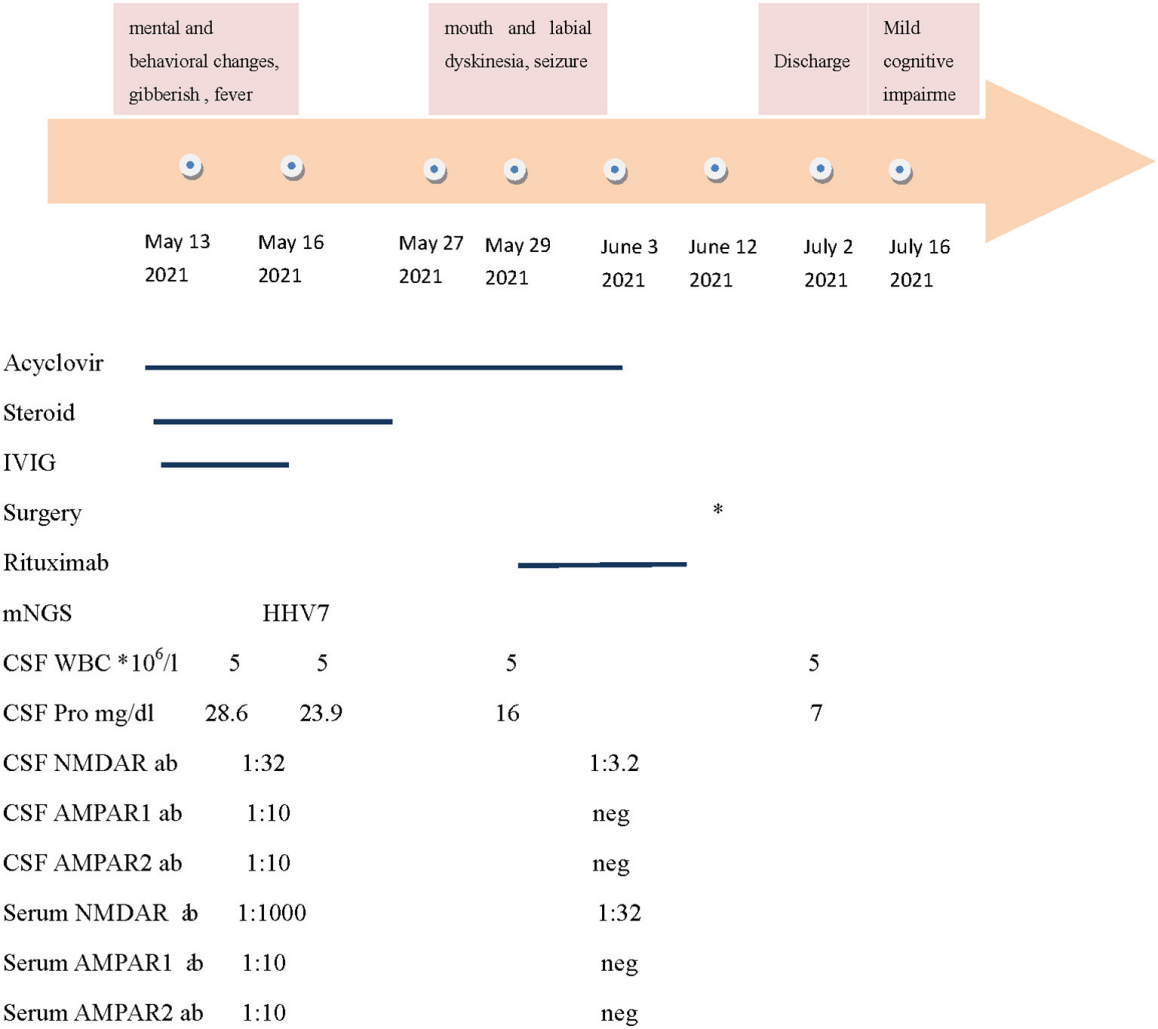
The summary of the clinical features, therapy, and antibody titers. NMDAR: N-methyl-Daspartate receptor; AMPAR: alpha-amino-3-hydroxy-5-methyl-4-isoxazolepropionic acid receptor; CSF: cerebrospinal fluid; and IVIG: intravenous immunoglobulin. * The patient underwent laparoscopic partial ovariectomy on June 12. Neg: negative.

A 36-year-old woman was admitted to the Department of Neurology at Affiliated Ganzhou Hospital of Nanchang University on May 13, 2021, due to mental and behavioral changes. She was gibberish for 13 days, followed by fever for 1 day (Figure 1). She had an unremarkable medical history with no prior medical conditions.

**Figure 2:**
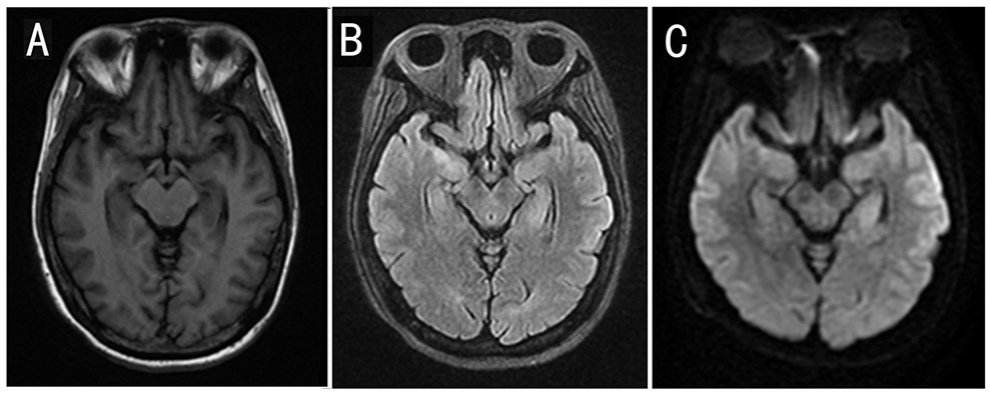
Cranial MRI results of the patient. Cranial MRI showed an increased signal intensity in the mesial temporal lobes and the hippocampus on T2 FLAIR and diffusion-weighted images. **(A)** T1-weighted image. **(B)** T2 FLAIR image. **(C)** Diffusion-weighted image.

Neurological exams on admission showed delirium, aphasia, extensor toe response, and Kernig's sign with the right side. Brain MRI revealed a fluid attenuation inversion recover (FLAIR) high-intensity lesion involving the mesial temporal lobes and the hippocampus on May 13, 2021 (Figure 2). Electroencephalogram (EEG) revealed a diffused slow wave. A lumbar puncture was performed on May 14, and the cerebrospinal fluid (CSF) analysis revealed a white blood count of 5 × 10^6^/L, protein of 28.6 mg/dl (normal range, <40 mg/dL), and glucose of 3.01 mmol/L (normal range, 2.8–4.5 mmol/L). The PACEseq metagenomic next-generation sequencing (mNGS) (Hugobiotech, Beijing, China) of CSF was performed to identify the pathogens on May 16. A total of 7 unique reads of HHV-7 was detected. Considering the probability of autoimmune encephalitis, an antibody testing of CSF using a cell-based assay (Kindstar Global company) was also applied on May 15. The anti-NMDAR (titer of 1:32), anti-AMPAR1 (titer of 1:10), and anti-AMPAR2 (titer of 1:10) antibodies were detected as positive. Serum studies were also performed, demonstrating positive anti-NMDAR (titer of 1:1,000), anti-AMPAR1 (titer of 1:10), and anti-AMPAR2 (titer of 1:10) antibodies but negative anti-leucine-rich glioma- inactivated 1, anti-contactin-associated protein-like 2, and anti-GABA-B antibodies. Lymphocyte classification revealed the CD3 + CD4 + cells of 164.43/μl (normal range, 550–1,440/μl), CD3 + CD8 + cells of 190.47/μl (normal range, 320–1,250/μl), and CD19 + cells of 136.41/μl (normal range, 90–560/μl). So, autoimmune encephalitis was also considered. Antiviral treatment with acyclovir (0.5 g per 8 h, intravenously, for 21 days) was initiated on May 14. At the same time, high doses of corticosteroids (methylprednisolone, 1 g per day, intravenously, for 5 days, 500 mg per day for 3 days, 250 mg per day for 3 days) and intravenous immunoglobulin (0.4 g/kg body weight for 5 days) were also administered for the autoimmune encephalitis on May 16. However, the clinical condition of the patient deteriorated, with yelling and agitation. After the treatment of sedative drugs, the patient calmed down. However, her mental and behavioral symptoms did not disappear. On May 27, 2021, mouth and labial dyskinesia and seizure occurred. Levetiracetam and sodium valproate were administered for antileptic treatment. The condition of the patient is still impaired after the first-line therapy. On May 29, 2021, a second-line treatment of rituximab (375 mg/m^2^ weekly for 3 weeks) was used for her autoimmune encephalitis. On June 2, the titer of anti-NMDAR antibody was decreased to 1:32 and 1:3.2 in serum and CSF, respectively, and the anti-AMPAR antibodies turned negative. The symptoms of the patient continued to improve.

**Figure 3:**
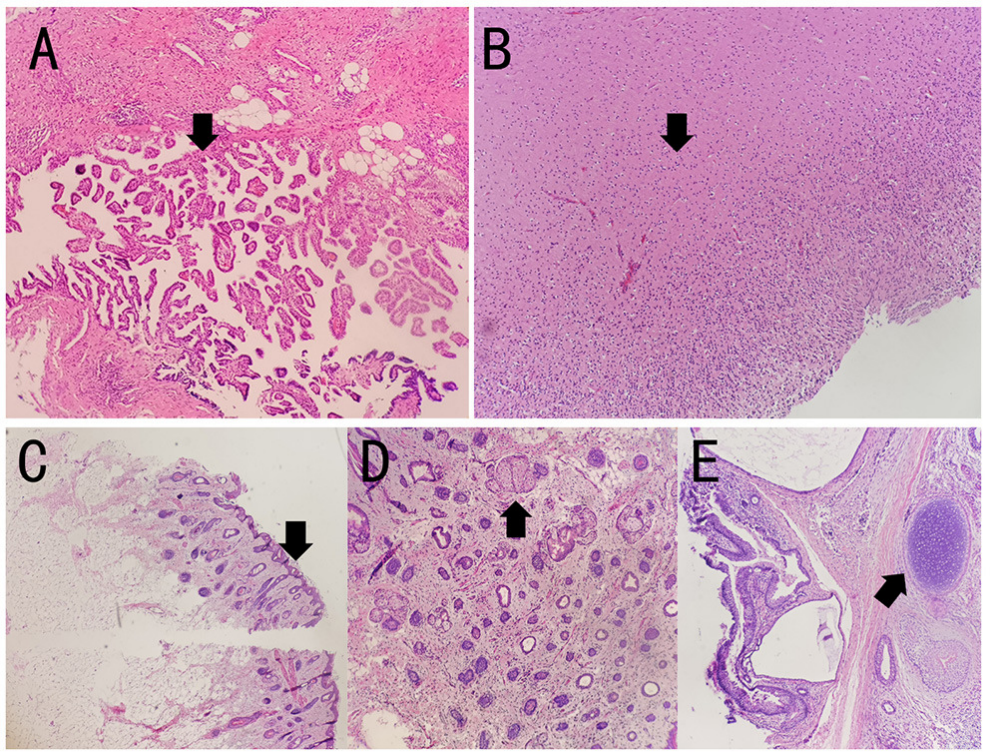
The pathology results of the patient. The specimen showed mature cystic teratoma, consisting of choroid plexus, neuropil, sebaceous glands, hair follicles, and cartilage tissue by H&E staining. The arrows indicate lesions in **(A–E)**. **(A–E)** Representative histopathological features of mature teratoma. **(A)** Choroid plexus. **(B)** Neuropil. **(C)** Hair follicles. **(D)** Sebaceous glands. **(E)** Cartilage tissue.

To evaluate the tumors, malignancy screening including a computed tomography (CT) of the chest, the abdomen, and the pelvic was tested, which demonstrated a pelvic mass of 4.8 ^*^ 4.2 ^*^ 4.9 cm, indicating ovarian teratoma. A smaller mass was also found and considered as a cyst. Subsequent pelvic MRI indicated an ovarian teratoma on June 11. Laparoscopic partial ovariectomy was then performed with tracheal incubation and mechanical ventilation on June 12. The pathology report showed mature cystic teratoma in the right ovarian, consisting of choroid plexus, neuropil, sebaceous glands, hair follicles, and cartilage tissue (Figure 3). Although with mild memory impairment and gibberish sometimes, the patient can speak simple words. Lymphocyte classification showed CD3 + CD4 + cells of 780.76/μl, CD3 + CD8 + cells of 756.91/μl, and undetectable CD19 + cells on July 15. She was discharged on July 16. On 1-month follow-up, despite mild memory impairment, she can perform all the usual duties and activities.

## Discussion

To the best of our knowledge, this is the first reported case of autoimmune encephalitis with multiple autoantibodies with concomitant HHV-7 infection and ovarian teratoma. No standard treatment for HHV-7-associated neurological complications has been established, although ganciclovir, foscarnet, and cidofovir are commonly used for HHV-7 antiviral treatment (13). Acyclovir is also an antiviral agent, which has a marked activity against the herpesviruses. It is reported that acyclovir is one of the most potent compounds with the highest anti-HHV-7 and can be used in central nervous system infection (14, 15). So, we empirically used acyclovir for this case with human herpesvirus encephalitis. The common clinical manifestations of anti-NMDAR and anti-AMPAR encephalitis included cognitive impairment, psychiatric disorder, dyskinesia, speech disorder, insomnia, autonomic dysfunction, and dysarthria (16). In this case, the patient's clinical manifestation of psychiatric disorder and cognitive impairment could be attributed to both anti-NMDAR antibody and anti-AMPAR antibody. Seizures and mouth and labial dyskinesia were associated with rgw anti-NMDAR antibody. After resection of ovarian teratoma and immunotherapy for autoimmune encephalitis, the related antibodies significantly decreased and even turned negative.

The HHV-7 rarely causes encephalitis, especially in immunocompetent patients. In a study conducted in Toronto, 57 (1.9%) out of 2,972 children were detected positive with HHV-7 DNA in their CSF, including 8 patients with meningitis and 7 patients with encephalitis (15). Clinical presentations of HHV-7 infection in the neurological system include a gradual loss of strength and weakness in the limbs, disorientation and confusion, flaccid paraplegia, localized anesthesia and hypoesthesia for pain and light touch, urinary retention, and constipation. These symptoms are similar to other encephalitides. So, it is hard to be successfully diagnosed by clinical practices. In addition, conventional diagnostic methods including culture and serology are not ideal for the detection of HHV-7. The mNGS is currently used in many infectious diseases and is performed with high sensitivity and specificity. It had a broad spectrum and can detect almost all pathogens. It has great advantages, for example, in identifying rare, novel, difficult-to-detect, and co-infected pathogens from clinical samples, especially for the detection of viruses (17). In this case report, HHV-7 was successfully identified by mNGS in CSF. The limitation of mNGS in this patient is that the HHV-7 DNA detection could not discriminate whether it is a primary infection or a latent reactivation, and it is complex to interpret the result. It has been reported that neurotropic viruses cause anti-NMDAR encephalitis by several mechanisms. One is that neurotropic viruses may lyse neurons, releasing antigens that stimulate antibodies to the NMDA receptor and triggering autoimmune encephalitis. Another proposed hypothesis is that anti-NMDAR encephalitis may stimulate latent virus reactivation, thus, leading to secondary encephalitis (8). To date, HHV-7 has not been documented in the setting of anti-NMDAR or anti-AMPAR encephalitis. The presentation is more of anti-NMDAR encephalitis than anti-AMPAR encephalitis, and AMPAR antibodies were at a low concentration (1:10 in CSF and serum). It may be a marker of increased auto-antibody production in response to a tumor cell breakdown, post-viral infection, and antigen release, or represent low-titer “false positive” results. Our patient completed the full first-line and second-line therapies for both anti-NMDAR and HHV-7 encephalitis, and her symptoms improved; it is unclear whether HHV-7 triggered the autoimmune condition of the patient, or it was present in the CSF as a result of latent viral reactivation. Her presenting symptoms were more consistent with anti-NMDAR encephalitis than HHV-7 encephalitis. Acyclovir treatment was used for 21 days because an immunosuppressive regimen for autoimmune encephalitis might worsen an underlying HHV-7 infection.

A recent study has reported that 4–7.5% of patients with anti-NMDAR encephalitis have concurrent glial antibodies or neuronal surface antibodies. Some of these antibodies have unique clinical features and may influence the prognosis (18). In that study, 6 patients had both anti-NMDAR and anti-AMPAR antibodies, most of whom were women (83%). About 67% of the patients had bilateral medial temporal lobe MRI abnormalities, and 83% had tumors (ovarian teratoma and breast cancer). All these patients had severe symptoms of anti-NMDAR encephalitis (4–5 groups of symptoms) with decreased levels of consciousness and required intensive care. In our case report, the patient was also a woman with severe symptoms of anti-NMDAR encephalitis (cognitive impairment, decreased level of consciousness, speech disorder, seizure, and dyskinesia), accompanied by a decreased level of consciousness and required admission to intensive care. Cranial MRI showed temporal lobe and hippocampal abnormality. Ovarian teratoma was found using cancer screening. Patients with co-existing anti-NMDAR antibody and neuronal surface antibody have a worse outcome than those with only anti-NMDAR antibody (19). Thus, early use of second-line immunotherapies is worth considering for these patients (18).

The limitation of the case is that HHV-7 DNA was only detected in the patient's CSF by mNGS. Serology and qPCR of HHV-7 in CSF and serum were not detected due to the limited amount of samples retained. The carrier frequency of HLA class II allele DRB1^*^16:02 was not detected in this paper either, which was reportedly associated with anti-NMDAR encephalitis (19).

In conclusion, a rare case of autoimmune encephalitis with multiple autoantibodies, concomitant HHV-7, and ovarian teratoma was found. These co-existing antibodies produced an overlap of clinical symptoms and lead to a worse prognosis of the patient. Thus, broad-spectrum etiological analyses, including multiple antibody panels, virus infections, and neoplastic risk factors, are needed for the evaluation of patients presenting with autoimmune encephalitis. Clinicians should consider concomitant therapy, especially in atypical cases. Additional pieces of research are needed to optimize the treatment regimen when the autoimmune encephalitis with multiple autoantibodies presents with concomitant viral detection.

## Data Availability Statement

The data of this manuscript is available at: http://ngdc.cncb.ac.cn, accession number: PRJCA007384.

## Ethics Statement

The studies involving human participants were reviewed and approved by Ethics Committee of Ganzhou People's Hospital. The patients/participants provided their written informed consent to participate in this study.

## Author Contributions

PW was the physician of the patient and collected the data for case presentation. JY participated in collecting data and drafted the manuscript. ZL reviewed the literature and participated in its design. XL reviewed the literature and performed infectious disease consultations. HX analyzed metagenomic next-generation sequencing data and contributed to language polishing. All the authors read and approved the final manuscript.

## Conflict of Interest

Author HX is employed by Hugobiotech Co., Ltd., Beijing, China. The remaining authors declare that the research was conducted in the absence of any commercial or financial relationships that could be conducted as potential conflict of interest.

## Publisher's Note

All claims expressed in this article are solely those of the authors and do not necessarily represent those of their affiliated organizations, or those of the publisher, the editors and the reviewers. Any product that may be evaluated in this article, or claim that may be made by its manufacturer, is not guaranteed or endorsed by the publisher.
